# The interplay of group size and flow velocity modulates fish exploratory behaviour

**DOI:** 10.1038/s41598-024-63975-z

**Published:** 2024-06-08

**Authors:** Gloria Mozzi, Daniel Nyqvist, Muhammad Usama Ashraf, Claudio Comoglio, Paolo Domenici, Sophia Schumann, Costantino Manes

**Affiliations:** 1 Department of Environment, Land and Infrastructure Engineering, Politecnico di Torino, Turin, Italy; 2 IBF-CNR Pisa, Institute of Biophysics, Pisa, Italy; 3 Institute for the study of anthropic impact and sustainability in the marine environment, IAS-CNR Oristano, Oristano, Italy; 4 Department of Biology, University of Padova, Padua, Italy

**Keywords:** Fish movement, Group behaviour, Hydrodynamics, Artificial intelligence, Deep learning, Freshwater ecology, Engineering

## Abstract

Social facilitation is a well-known phenomenon where the presence of organisms belonging to the same species enhances an individual organism’s performance in a specific task. As far as fishes are concerned, most studies on social facilitation have been conducted in standing-water conditions. However, for riverine species, fish are most commonly located in moving waters, and the effects of hydrodynamics on social facilitation remain largely unknown. To bridge this knowledge gap, we designed and performed flume experiments where the behaviour of wild juvenile Italian riffle dace (*Telestes muticellus*) in varying group sizes and at different mean flow velocities, was studied. An artificial intelligence (AI) deep learning algorithm was developed and employed to track fish positions in time and subsequently assess their exploration, swimming activity, and space use. Results indicate that energy-saving strategies dictated space use in flowing waters regardless of group size. Instead, exploration and swimming activity increased by increasing group size, but the magnitude of this enhancement (which quantifies social facilitation) was modulated by flow velocity. These results have implications for how future research efforts should be designed to understand the social dynamics of riverine fish populations, which can no longer ignore the contribution of hydrodynamics.

## Introduction

Social facilitation refers to the phenomenon in which an individual's performance in a particular task is enhanced by the presence of conspecifics^1^. It has been extensively studied in diverse organisms, ranging from insects to birds and mammals^2–4^, highlighting its importance in understanding social dynamics and ecological processes. Fish, with their varied social structures and diverse behaviours, offer a fascinating opportunity to explore social facilitation in different contexts. Among gregarious species, fish can display social facilitation in relation to foraging^5,6^, predator avoidance^7,8^, and exploration^9–12^. For example, groups of minnows (*Phoxinus phoxinus*) and goldfish (*Carassius auratus*) exhibited a reduction in duration and frequency of hiding behaviour as group size increased during foraging (2, 4, 6, 12, and 20 individuals)^9^. Interestingly, this group size effect was more marked on minnows than goldfish, possibly due to the higher schooling tendency of the former species. A study on three-spined sticklebacks (*Gasterosteus aculeatus*) reported that groups exhibited higher activity levels and demonstrated quicker resumption of foraging when exposed to simulated predation risk compared to a fish alone^11^. Another experiment showed that when young-of-the-year perch (*Perca fluviatilis*) were tested in single versus four-fish groups, the fish in groups spent more time exposed and approached areas close to a predator for feeding more quickly than single fish^12^.

Although the energetic benefits of schooling are widely recognised^13–16^, literature on social facilitations has typically focused on fish in standing waters^5–12^. In riverine environments, standing water represents only a limited portion of available habitats, and, therefore, very little is known about how social facilitation affects riverine fish species. A handful of studies hints that hydrodynamics might play an important role in the dynamics of fish groups (reviewed in^17^, reporting that flow velocities can regulate the structure and polarization of fish schools^18^^,^ which, in turn, can affect information transfer among individuals^19^. Another limitation in the existing literature relates to the fact that most of the past experimental studies were performed either by forcing fish to swim in very shallow waters^20^ or by neglecting their activity in the water column without thoroughly validating the exclusivity of horizontal motion^10,21–23^. This not only hampers the interpretation of results within the horizontal plane but also overlooks fish’s spatial utilisation of the water column. Recognising how fish shoals navigate the available space in flowing waters is important for understanding their social dynamics, including the exploitation of boundaries for energy saving.

This lack of knowledge about how hydrodynamics affects social facilitation represents a bottleneck to understanding the dynamics of fish populations in riverine environments. Here, we focus on how being in a group influences the exploratory behaviour of individual fish and, in turn, how this is affected by hydrodynamics. This is relevant because exploratory behaviour is key to acquiring vital resources such as food, shelter, and mating partners while also facilitating the acquisition of other valuable information about their surroundings, including the presence of predators and environmental changes^24^. From a practical standpoint, this holds relevance for developing efficient management strategies related to river flow regulation, restoration efforts, and the design of fish passage structures^25^. This is particularly crucial in light of the severe decline observed in migrating fish populations, often attributable to river fragmentation^26^.

The aim of the present study is to investigate the role and interplay of social facilitation and mean flow velocity in dictating the exploratory and swimming behaviour of riverine fish. To accomplish this goal, we conducted flume experiments on groups of wild juvenile Italian riffle dace (*Telestes muticellus*) swimming in open channel flows. We hypothesise that (i) group size increases the explored area (hereafter referred to as EA) and the swimming trajectory length (hereafter referred to as TL) of individual fish, meaning that social facilitation takes place and (ii) the magnitude of this response is affected by flow velocity. Additionally, we investigated the preferred swimming areas, referred to as space use, to enrich the interpretation and discussion of results pertaining to EA and TL.

## Materials and methods

### Experimental set-up

**Figure 1:**
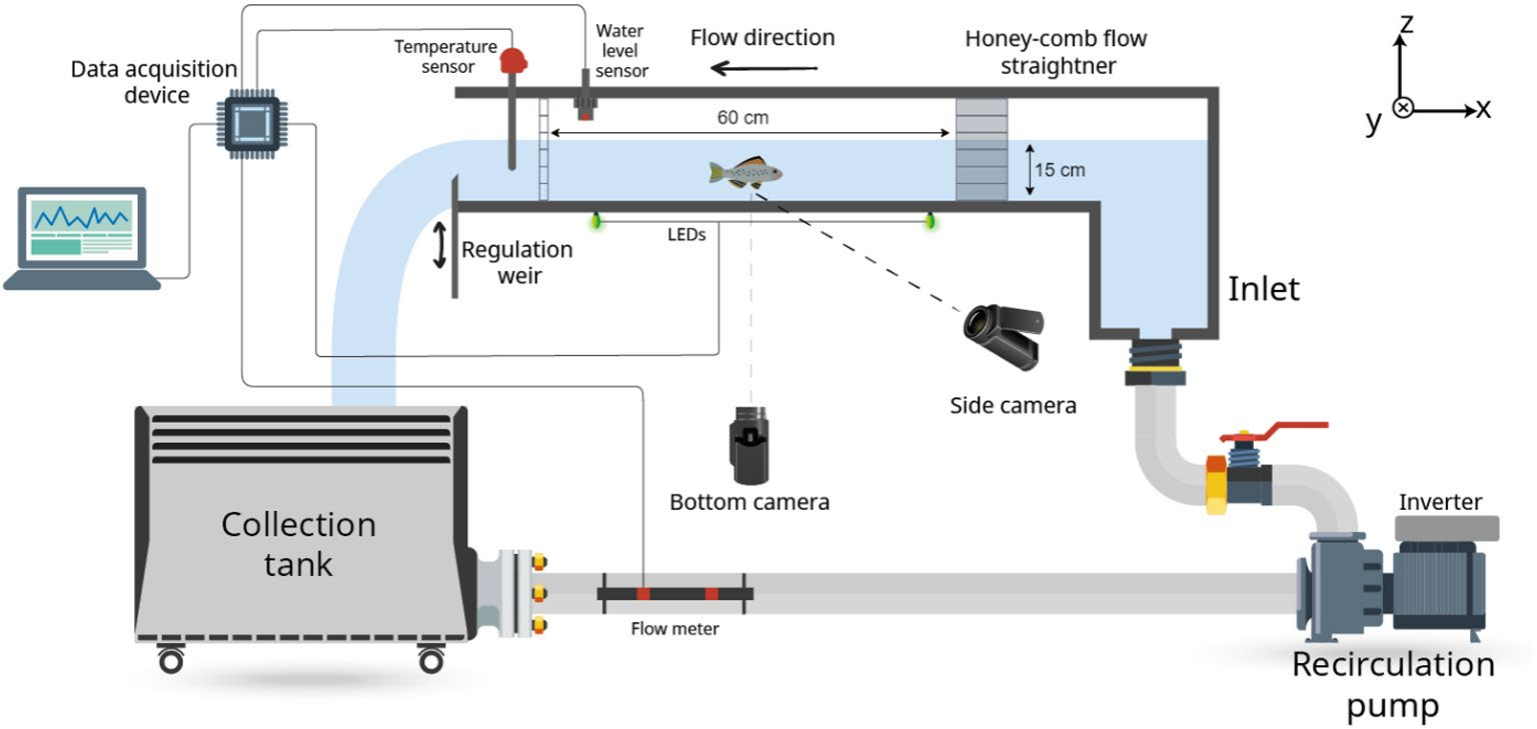
Schematic representation of the experimental set-up. The test section dimensions were 30 cm (width, not shown) × 60 cm (length) × 15 cm (depth), as depicted in the diagram. Tripods (not shown) were used to position cameras: one beneath the flume and another at the middle depth of the test section (i.e. 7.5 cm above the bottom of the flume).

Experiments were conducted in a hydraulic flume (150 cm × 30 cm × 30 cm in length, width and height, respectively) with water supplied by a recirculation pump connected to a downstream storage tank via a series of pipes (Fig. 1). Flume walls were made of transparent Perspex with an aluminium frame. The test section was delimited upstream by a honeycomb aluminium flow straightener and downstream by a movable stainless-steel grid with a mesh size of 0.5 cm × 0.5 cm. The test section was reduced to a volume of 60 cm × 30 cm × 15 cm (longitudinal, lateral and vertical directions, respectively); its length could be regulated by moving the downstream grid, while the pump frequency (controlled with an inverter) and the height of a downstream sharp-crested weir controlled the flow rate and depth.

A PT100 thermoresistance was used to measure the temperature of the water, and a 440 W chiller regulated the temperature. A flowmeter (AquaTransTM AT600, Baker Hughes) installed on the steel pipe at the bottom of the collection tank measured the flow rate, while the water level was measured 5 cm upstream of the downstream grid with an ultrasonic sensor (BUS0025, BALLUFF). A data acquisition device (DAQ USB-6002, National Instruments) collected and transferred data to a personal computer for logging. The mean flow velocity – retrieved from the flow rate and water leve–was consistently monitored on the computer from a LabVIEW interface (National Instruments). The data acquisition device also allowed for the control of three LED diodes installed beneath the flume within the camera view, used to synchronise video recorded from multiple cameras.

To characterise the hydrodynamic conditions of the investigated flow and consequently ensure the replicability of the experimental campaign, we performed velocity measurements by means of Lased Doppler Anemometry (LDA). Such measurements, which provide only a patchy description of the hydrodynamics of our experiments, were then used to fine-tune computational fluid dynamics (CFD) simulations, which instead provide a more complete description of the flow. The outcomes of this characterisation are outlined in Sect. 5 “Hydrodynamic characterization of the flume” within the Supplementary Material.

### Fish capture and holding

Italian riffle dace (*Telestes muticellus*) is a small-sized (< 15 cm), omnivorous, gregarious fish native to streams and rivers on the Italian peninsula^27^. About 100 juvenile Italian riffle dace of a mean length of 5.14 cm (SD ± 0.34 cm) were captured through electrofishing in Noce stream (44° 56′ 18.52″ N 07° 23′ 11.24″ E) in northern Italy. Fish were then taken to a nearby hatchery and placed in flow-through tanks. After one week, fish were transferred to an artificial pond fed by river water with an average temperature of 15.4 ℃ (SD ± 0.9 ℃). Here, they could feed ad libitum thanks to the organic matter incoming from the stream.

The fish were relocated to three separate tanks (each of 200 × 60 × 15 cm^3^) located inside the hatchery and supplied with spring water at 13.6 ℃ (SD ± 1 ℃) 90 h before the start of the experiments to acclimate for hatchery conditions. Each tank was divided into three compartments, with dimensions of 60 × 60 × 15 cm^3^, hosting less than 20 fish per compartment. Opaque plastic panels were placed on top of the tanks to provide a protective covering against potential disturbance caused by passing operators. Experiments were conducted in June 2021, and water temperatures in the hatchery tanks and the outside pond were continuously monitored throughout the experimental period.

### Experimental protocol

Randomised experiments were conducted on three different group sizes (one, two, and six fish, refer to Supplementary Material, Sect. 3 “Randomisation” for details on the randomisation procedure). All fish underwent exposure to a series of mean flow velocities: 10, 20, and 35 cm/s. These values were chosen to represent velocity regimes that ranged from non-challenging (10 cm/s) to moderately challenging (20 cm/s) and, finally, more challenging (35 cm/s) for the fish. This velocity range was selected based on the *maximum sustained speed* (${U}_{ms}$), which was determined using the Videler equation (${U}_{ms}=$ 27 cm/s for fish of body length 5 cm)^28^. Flow velocities were tested sequentially rather than independently, as testing them separately would have necessitated an impractical sample size of fish (more than 300) to maintain statistical robustness. This quantity exceeded what was considered reasonable to extract from the river. The experimental campaign comprised a total of 100 fish: 20 trials for single fish (i.e. 20 fish), 10 trials for two-fish groups (i.e. 20 fish), and 10 trials for six-fish groups (i.e. 60 fish), with each fish tested only once. Each group was exposed to three consecutive mean flow velocities: 10 cm/s for 15 min (5 min for acclimation^29^ and 10 min for the actual trial), 20 cm/s for 10 min, and 35 cm/s for 10 min. The test section was delimited to a length of 60 cm (i.e. about 12 body lengths), while water depth was kept at 15 cm (i.e. about three fish body lengths). Water temperature was maintained at 14 ℃ (SD ± 0.3 ℃) throughout the experiments.

If a fish rested at the downstream grid, it was gently tapped from behind with a rod, but no more than three times per group within the same velocity treatment^29^. Given that most fish could swim throughout the entire experimental period after tapping, we inferred that this behaviour was indicative of an initial reluctance to swim rather than fatigue. We considered potential behavioural anomalies resulting from tapping comparable to stress induced by the unfamiliar environment and capture-and-release process. After each trial, the fish were anaesthetised in clove oil (Aromalabs, USA,approximately 0.05 ml clove oil/l water) and measured for weight and fork length. Some individuals underwent physiological analysis, assessing cortisol and oxidative stress levels in muscle tissue. The complete procedure and results can be found in Schumann et al.^30^.

### Video tracking

The experimental trials were recorded from the side and bottom using two video cameras (Sony FDR-AX43; 1920 × 1080 pixels, 50 fps, see Fig. 1). LED diodes were switched on at the beginning of each trial to provide a datum for time and ensure that videos from different cameras could be later synchronised. The analysis did not include the first three minutes of each 10 min trial to give the fish time to adapt to the new water velocity. Fish trajectories were reconstructed following two steps: detection and training.

Detection involved a deep learning approach with a convolutional neural network (CNN) to identify fish heads in both bottom and lateral video footage (YOLOv4^31^). To this end, two networks were trained—one for the bottom and one for the side videos, each with approximately 10 000 annotated images (for further details, see Sect. 1.1 “Training” in the Supplementary Material). The resulting F1 scores for the bottom and side CNNs were 96% and 91%, respectively (refer to Supplementary Material, Sect. 1.2 “Testing CNN performances” for details). Each CNN provided bounding boxes that enabled the detection of fish heads in each frame, with each detection providing the coordinates of the centroid of the bounding box, as well as its height, width, and confidence level [0, 1] indicating the accuracy in representing a fish head, determined by the network.

For the horizontal coordinates, a custom algorithm developed in Matlab^32^ was used to convert CNN detections (i.e. positions of the identified fish in single video frames) into tracking trajectories (i.e. the path taken by each fish over time through the video sequence). This algorithm was based on a Kalman filter approach^33^^,^ which embedded an identity recognition routine employing a stochastic approach to assign fish identity in videos featuring two or six fish. This routine systematically analysed all possible combinations of fish trajectories between two consecutive frames and selected the solution that minimised the overall group acceleration during this interval. This relatively straightforward method was developed for its compatibility with integration into the Kalman filter and its computational efficiency, particularly for our most data-intensive videos (i.e. six-fish groups,for detailed information, refer to the Supplementary Material, Sect. 2 “Tracking”). The algorithm parameters were set to maximise tracking performance during occlusions (i.e. frames in which one or more fish in a video sequence become partially or entirely obscured by other objects or the environment). The performance of the tracking algorithm was validated using a randomly selected sample of 2 min of videos featuring six-fish groups. By comparing the algorithm’s output with a visual check conducted by an operator, it was determined that the algorithm accurately assigned identity in 87% of occlusions (see Supplementary material, Sect. 2.6 “Validation”).

Regarding the vertical coordinates, the primary goal of the side videos was to assess to what extent fish swam away from the flume bottom. Although CNN exhibited high performance, videos featuring six-fish groups were challenging to analyse from the lateral plane. This was primarily due to frequent occlusions, where fish would temporarily obstruct each other within the camera’s field of view. As a result, there were instances of missing detections (MD) in these videos, and after a quantitative analysis of this occurrence (see Supplementary Material, Sect. 1.3 “Detection along the vertical coordinate”), side videos were considered unsuitable for the tracking phase. Nevertheless, position data on the vertical plane were analysed from CNN detections for assessing vertical space use, only without tracking the individual trajectories.

### Data analysis

A reference system was defined with the origin at the honeycomb's bottom-right corner of the flume (upstream end of the experimental arena). The x-axis was aligned with the flow direction, the y-axis was set as transversal to the water flow in the horizontal plane, and the z-axis was defined as the vertical coordinate. Units of fish position resulting from the video analysis were converted from pixels to centimetres in the defined reference system using the coordinates of the four corners of the swimming arena for each video, as described in Nyqvist et al.^34^.

Data analysis was conducted using R v4.0.5^35^. Fish positions obtained from video-tracking were utilised to compute the following metrics: (i) explored area (EA), (ii) trajectory length (TL), and (iii) space use, with the first two only analysed in the horizontal plane $(x,y)$ – as vertical space use revealed that fish mostly stayed close to the flume bottom (see “Results” section). EA was defined as the group-average ratio of the area covered by each fish to the total horizontal arena available. To calculate EA, a 60 × 30 cm grid representing the experimental arena was utilised, and the cumulative count of newly covered 1 cm^2^ cells by each fish along its path was determined and normalised by the total number of cells in the grid (i.e. 1800). Subsequently, the EA was calculated as the average of this count among all the fish in the investigated group. TL was determined as the horizontal distance swam by fish within a trial relative to the previously defined stationary reference system. Like EA, TL was calculated by averaging the trajectories of all fish in the group. Finally, space use was defined using two metrics: the first was ${P}_{H}(x,y)$, representing the probability density associated with a fish occupying a specific horizontal position $(x,y)$. The second was ${P}_{V}\left(z\right)$, which represents the probability density of the vertical position of fish along the normal coordinate $z$. Both metrics were calculated utilising fish positions from all experiments conducted under the respective velocity and group size conditions. ${P}_{H}$ and ${P}_{V}$ were retrieved using a Kernel density estimation approach^36^ with a fixed bin width of 1 cm for discretisation of both ${P}_{H}$ and ${P}_{V}$ in all spatial coordinates.

Plots of ${P}_{H}$ and ${P}_{V}$ were generated using the *geom_density_2d* and *ggridges* commands in the *ggplot2* v.3.4.0^37^ package in R, respectively. In the case of ${P}_{H}$, a logarithmic scale was employed to enhance the differences between trials. Estimating ${P}_{V}$ posed challenges due to the numerous occlusions observed from the side camera’s perspective. To derive ${P}_{V}$, a weighing approach was employed based on the total detections of each experiment. However, it is important to note that this approach led to an overestimation of ${P}_{V}$ as group size increased. The overestimation was a consequence of fish swimming closely together at the bottom of the flume, resulting in overlapping within the lateral camera view. See Supplementary Material for more details (Sect. 1.3 “Detection along the vertical coordinate”).

The statistical analysis was conducted using the *rstatix* (v0.7.0^38^ package in R with a significance threshold set at a p-value of 0.05. Space use medians of probability density functions $\widetilde{{\text{P}}_{H}}\left(x\right),\widetilde{{\text{P}}_{H}}\left(y\right)$ and $\widetilde{{\text{P}}_{V}}\left(z\right)$ were employed to characterise the overall positioning of fish along all three spatial coordinates (${P}_{H}(x)$ and ${P}_{H}(y)$ are the marginal distributions of ${P}_{H}(x,y)$ and the tilde symbol stands for median of a distribution)*.* For all analysed variables, each velocity treatment resulted in a sample size of N =[20,10,10] for [1,2,6] group sizes, respectively.

Data from EA, TL, $\widetilde{{\text{P}}_{H}}\left(x\right),\widetilde{{\text{P}}_{H}}\left(y\right)$ and $\widetilde{{\text{P}}_{V}}\left(z\right)$ did not fulfil the assumptions for parametric tests. Consequently, non-parametric tests were employed to examine the impact of group size and velocity on these variables. To test the hypothesis that social facilitation influenced EA and TL, we used the one-sided Jonckheere-Terpstra trend test, expecting larger effects for larger group sizes^39,40^. Effects of group size on EA and TL were tested for each velocity separately. With regards to the effect of group size on space use (i.e. $\widetilde{{\text{P}}_{H}}\left(x\right),\widetilde{{\text{P}}_{H}}\left(y\right)$ and $\widetilde{{\text{P}}_{V}}\left(z\right)$), our focus was directed towards detecting differences rather than trends. As a result, we utilised the Kruskal–Wallis test for these variables^41^. The Friedman test was employed to examine the effect of flow velocity (within-subject factor), taking repeated measures across flow velocities into account^42^. In cases of significant effect of velocity from the Friedman test, we conducted post-hoc analyses using paired Wilcoxon signed-rank test^43^. To account for multiple comparisons, we adjusted p-values of post-hoc tests using the Bonferroni correction method^44^.

A bottom-filtered analysis of EA, TL, ${P}_{H}$ was conducted to ensure the representativeness of the horizontal analysis results despite fish movement within the water column. This analysis exclusively considered frames where no fish was detected above 5 cm from the flume bottom. Additional information regarding the methodology and outcomes of the bottom-filtered horizontal analysis can be found in the Supplementary Material (Sect. 4, “Bottom-filtered horizontal analysis”).

### Ethical approval

The study was conducted following the Declaration of Helsinki and in accordance with the ARRIVE guidelines^45^. The study protocol was approved by the Protection of Flora and Fauna Department of the Metropolitan City of Turin (authorized by D.D. n.4457 of 29 October 2020), and conducted in accordance with the relevant guidelines and legislation under the provisions of art.2 of the national Decree n.26/2014 (implementation of Dir. 2010/63/EU). The study was conducted in accordance with relevant guidelines and legislation.

## Results

### Explored area and trajectory length (EA and TL)

**Figure 2:**
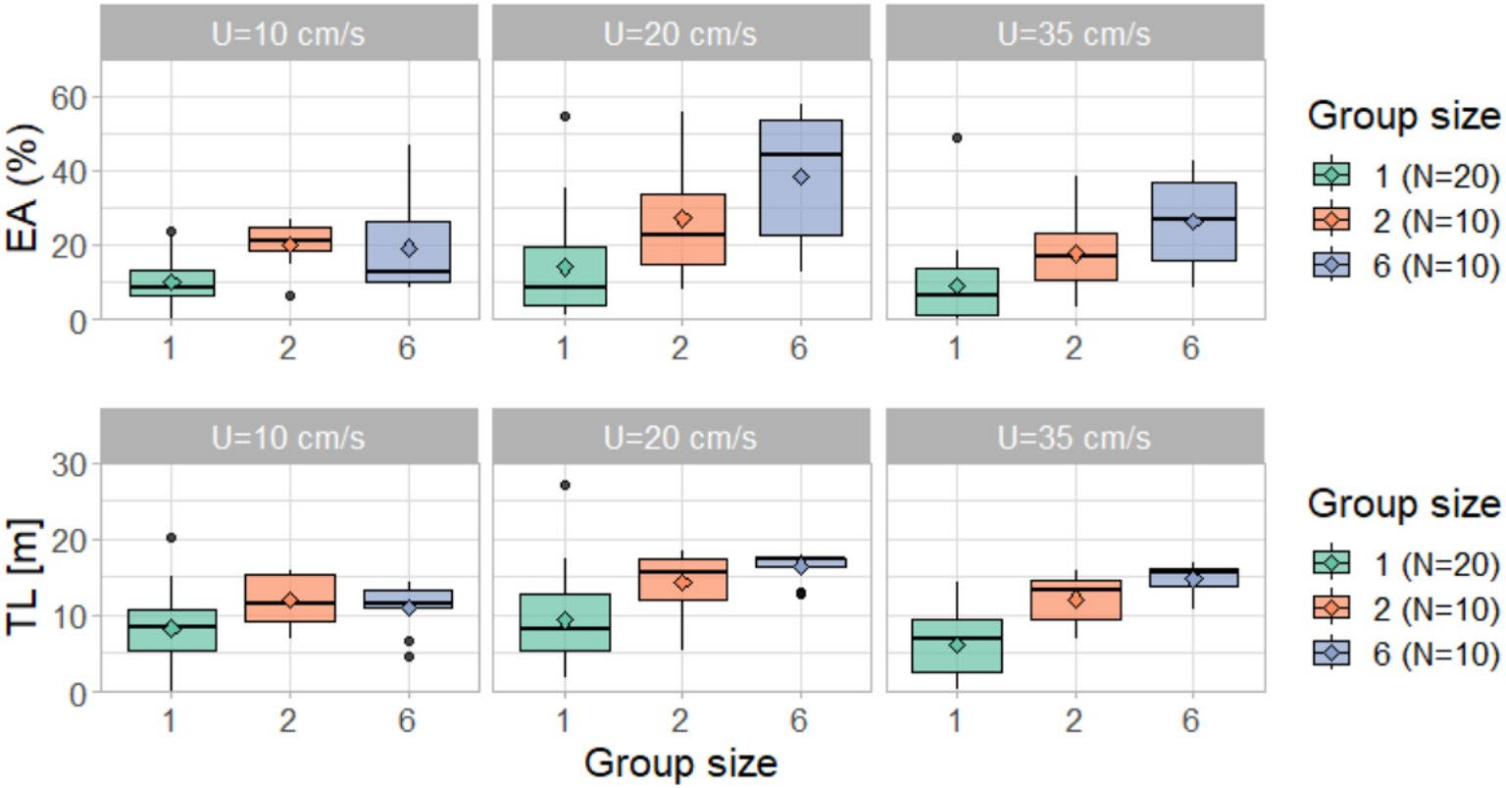
Explored area (EA, top) and trajectory length (TL, bottom) for each fish in the horizontal plane. In the case of two and six-fish groups, values represent the group-average. The diamonds represent the mean values.

The majority (75%) of fish displayed an EA lower than 20% of the total available horizontal arena and a TL lower than 13 m when alone in the flume (Fig. 2). By contrast, fish belonging to groups of six had an average EA of 19% (SD ± 13%), 38% (SD ± 18%), and 26% (SD ± 12%) at 10, 20, and 35 cm/s, respectively. Most (75%) of individuals of the six-fish group had a TL higher than 13 m at medium and high velocities, but at low velocity, they swam on average 11 m (SD ± 5 m). Fish swimming in pairs displayed an intermediate behaviour, with an average EA of 20% (SD ± 7%), 27% (SD ± 17%), and 17% (SD ± 11%) for low, medium, and high velocity, respectively. The average TL of fish in groups of two was 12 m (SD ± 4 m) at 10 cm/s, 14 m (SD ± 5 m) at 20 cm/s and 12 m (SD ± 5 m) at 35 cm/s (Fig. 2). Overall, fish displayed the highest EA and TL in groups at medium and high velocities, while group size had no effect at lower flow conditions.

The Jonckheere-Terpstra test revealed a significant trend with group size on EA and TL only for the medium and high velocities. At a velocity of 10 cm/s, group size did not display a significant trend on EA (z = 1.40, p-value > 0.05) or TL (z = 0.99, p-value > 0.05). By contrast, at the velocity of 20 cm/s, group size significantly increased EA (z = 3.27, p-value < 0.001) and TL (z = 3.42, p-value < 0.001). At the maximum investigated velocity, 35 cm/s, trends in group size were significant for EA (z = 3.49, p-value < 0.001) and TL (z = 4.33, p-value < 0.001).

Friedman test showed that in fish belonging to groups of six, flow velocity exhibited a significant effect on both EA and TL (χ^2^(2) = 11.4, p-value = 0.007 for EA, and χ^2^(2) = 20, p-value < 0.001 for TL). For single fish, instead, velocity only showed significance for EA (χ^2^(2) = 6.46, p-value < 0.05) and not for TL (p-value > 0.05). Notably, in two-fish groups, velocity did not play a significant role in either EA or TL (p-value > 0.05). Post-hoc analysis revealed that flow velocity had a significant effect on EA for single fish between medium and high velocities (20 and 35 cm/s, respectively, Table 1). For six-fish groups instead, the effect on EA was significant in the transition between low and medium flows (10 and 20 cm/s, respectively), while the effect on TL was significant comparing all velocity conditions.

**Table 1:** Post-hoc analysis on the effect of flow velocity on explored area (EA) and trajectory length (TL) with paired Wilcoxon test with Bonferroni adjustment for p-values.

Cluster	Pairwise comparison	EA	TL
Single fish	10 vs. 20 cm/s	NS	(–)
	10 vs. 35 cm/s	NS	(–)
	20 vs. 35 cm/s	0.034	(–)
Two fish groups	10 vs. 20 cm/s	(–)	(–)
	10 vs. 35 cm/s	(–)	(–)
	20 vs. 35 cm/s	(–)	(–)
Six fish groups	10 vs. 20 cm/s	0.008	0.006
	10 vs. 35 cm/s	NS	0.002
	20 vs. 35 cm/s	NS	0.004

For clusters in which the Friedman test did not show significance, post-hoc analysis was not computed (–).

NS stands for non-significant (p-value ≥ 0.05).

### Space use (${{\varvec{P}}}_{{\varvec{H}}},{{\varvec{P}}}_{{\varvec{V}}}$)

**Figure 3:**
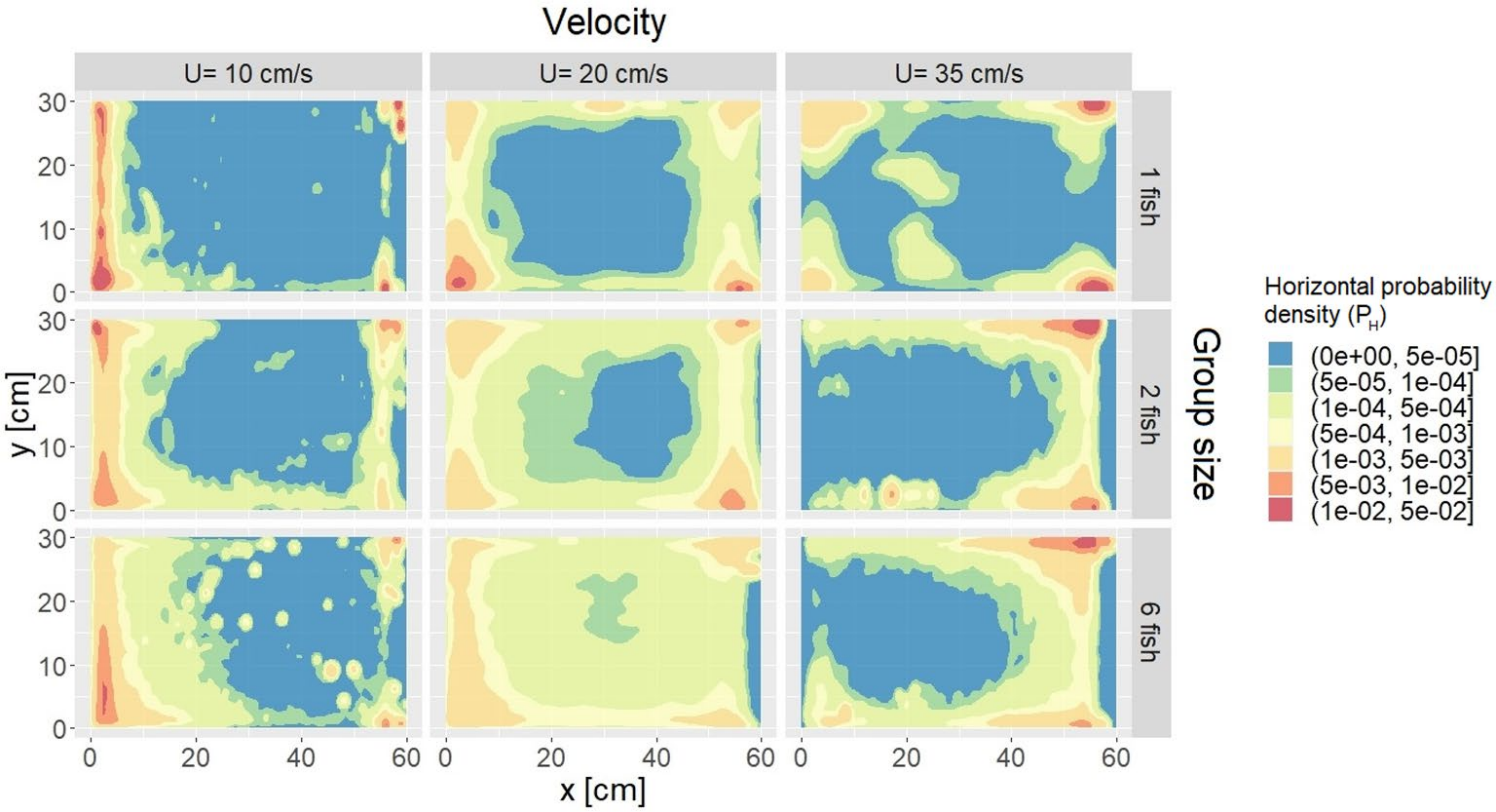
Horizontal probability density (${\text{P}}_{\text{H}}$) illustrating fish distribution at various flow velocities (10, 20, and 35 cm/s) and group sizes (1, 2, and 6). ${\text{P}}_{\text{H}}$ is obtained through Kernel density estimation, utilising fish positions (x, y) from all experiments conducted under the respective velocity and group size conditions. In each panel, water is flowing from left to right.

**Figure 4:**
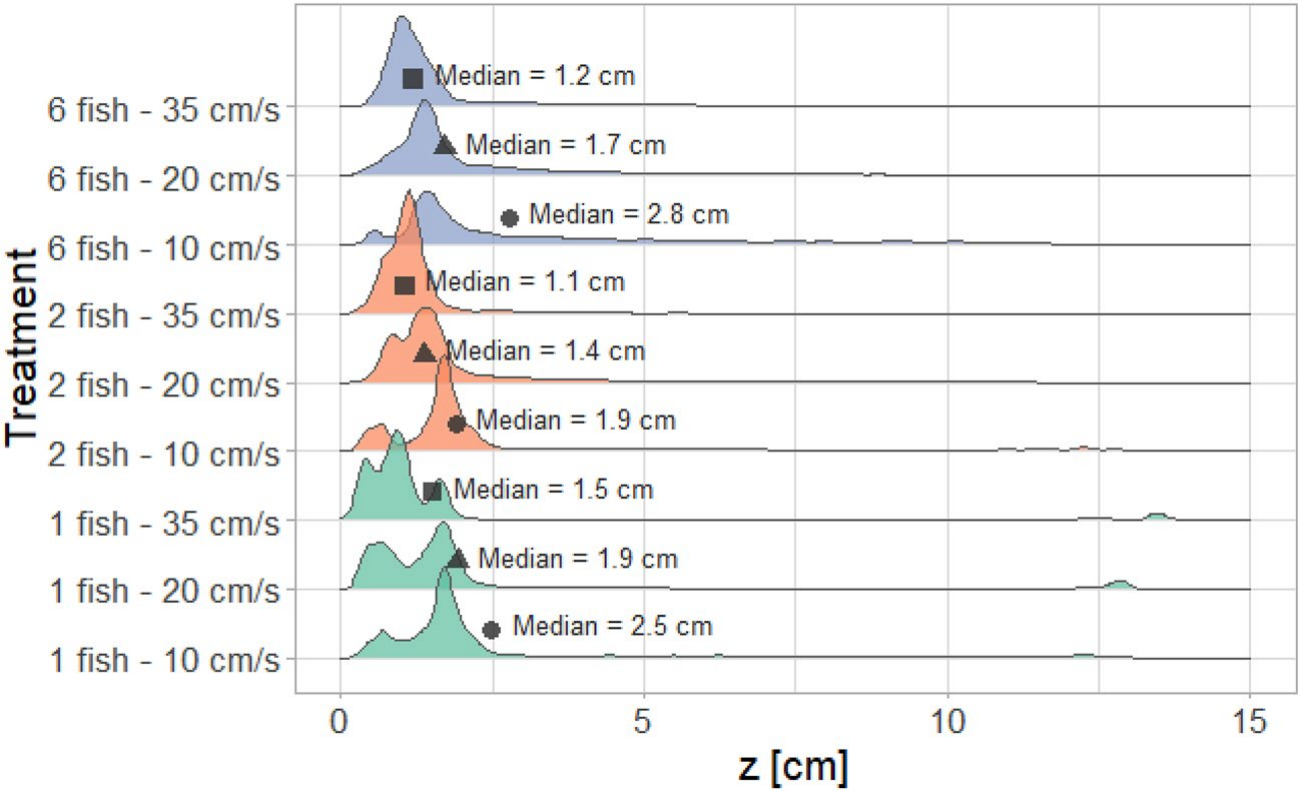
Vertical probability density (${\text{P}}_{\text{V}}$) for different group sizes and velocities (bottom of the flume: $\text{z}$=0; free water surface: z = 15 cm). Each P_V_ is retrieved with Kernel density estimation of all vertical fish positions detected at the respective flow velocity and group size.

Horizontal probability density, ${P}_{H}$, revealed that at the lowest velocity, fish tended to stay close to the upstream honeycomb grid while they relocated towards the downstream end of the flume and closer to the side walls as flow velocity increased (Fig. 3). For similar velocities, all group sizes tended to occupy similar areas. However, larger groups occupied a broader area compared to smaller groups at all three flow velocities, and the central region was sparsely occupied, particularly for single fish. Fish occupied the largest area at the medium velocity (20 cm/s) for all group sizes. As far as vertical space use ${P}_{V}$ is concerned, fish mostly remained within the first 5 cm from the flume bottom and swam closer to the bottom as velocity increased (Fig. 4). Notably, the vertical analysis underestimated the actual number of fish at the bottom of the flume (see Supplementary Material, Sect. 1.3 “Detection along the vertical coordinate”). Velocity had a significant effect (p-value = 0.023) on the longitudinal and vertical median positions (identified as $\widetilde{{\text{P}}_{H}}\left(x\right)$ and $\widetilde{{\text{P}}_{V}}\left(z\right)$, respectively) of the fish for all group sizes (Table 2). In contrast, lateral median position ($\widetilde{{\text{P}}_{H}}\left(y\right)$) was only affected by velocity for six-fish groups. Group size did not significantly affect the median position of fish (p-value > 0.05).

**Table 2:** Effect of group size and velocity on medians of probability distributions ($\widetilde{\text{P}}$) in the three coordinates (x, y, and z representing longitudinal, lateral and vertical positions, respectively).

Factor	Cluster	$\widetilde{{P}_{H}}(x)$	$\widetilde{{P}_{H}}(y)$	$\widetilde{{P}_{V}}(z)$
Group size^1^	10 cm/s	NS	NS	NS
	20 cm/s	NS	NS	NS
	35 cm/s	NS	NS	NS
Velocity^2^	Single fish	0.033	NS	< 0.001
	Two-fish group	0.005	NS	0.011
	Six-fish group	< 0.001	0.029	< 0.001

The effect of group size (between-subject factor) was assessed with Kruskal–Wallis test, while the impact of velocity (within-subject factor) was analysed through Friedman test (df = 2 for all tests).

NS stands for non-significant (p-value ≥ 0.05).

^1^Between-subject factor: Kruskal–Wallis test.

^2^Within-subject factor: Friedman test.

## Discussion

Overall, the results demonstrate that individuals within groups exhibited higher exploratory behaviour (i.e. higher EA) and swimming activity (i.e. higher TL) compared to fish swimming alone and that these increments were found to be affected by flow velocity. Preferred swimming areas–denoted by horizontal and vertical space use (${P}_{H}$ and ${P}_{V}$, respectively)–were mainly governed by flow velocity and not by group size. We observed that fish spontaneously swam in the near-bottom region, and the horizontal bottom-filtered analysis revealed no discernible differences in the primary trends of EA, TL, and ${P}_{H}$ when compared to the unfiltered data (refer to the Supplementary Material, section 4 “Bottom-filtered horizontal analysis”). Thus, we could reasonably assume that the estimation of EA, TL, and ${P}_{H}$ through a two-dimensional reconstruction of trajectories was highly representative of fish behaviour within the entire volume.

In order to make them feasible, treatments were conducted consecutively with an increase in flow velocity within a trial. This carries a potential time dependency, where higher velocities always occur after a longer time in the flume. Even though it cannot be definitely established, there are good reasons to believe that the observed effect of velocity is real and not a hidden effect of time in the flume. Previous studies on guppies (*Poecilia reticulata*) and mosquitofish (*Gambusia holbrooki*), revealed that the influence of time on exploratory behaviour becomes significant after 3^46^ and 4 hours^10^, respectively. Fish are also unlikely to fatigue under the time and velocities of the experiment. Most of similar-sized *Telestes muticellus*, were able to swim for longer than 30 min at 35 m/s in a dedicated (single fish) fatigue test^47^. Given this and the relatively short duration of our experimental trial (35 min in total), it is reasonable to infer that flow velocity exerted a more pronounced influence within the studied timeframe compared to the role of time itself, which has been reported to show significant effects only after longer durations (180 and 240 min according to Refs.^46^ and^10^, respectively).

Horizontal density distribution showed that locations of high fish density areas were unaffected by group size but were significantly influenced by velocity (Table 2). As flow velocity increased, fish moved towards the downstream grid and near-wall regions (Fig. 3). On the vertical plane, they positioned themselves closer to the flume bottom as velocity increased, regardless of group size (Fig. 4). It is interesting to note that flow velocity played a role in fish horizontal and vertical positioning (${P}_{H}$ and ${P}_{V}$, respectively). These space use patterns are likely driven by the need to seek less challenging flow conditions, as local velocities near the walls or bottom boundary layer are typically lower than in central regions or the intermediate layers of the water column^48,49^.

The increase of exploration and swimming activity with increasing group size is a form of social facilitation, intended as the promotion and enhancement of exploration and swimming activity in the presence of conspecifics^9–12^. This type of social facilitation is reported in the literature for other species in standing water, including minnows (*Phoxinus phoxinus*)^9^, goldfish (*Carassius auratus*)^9^, perch (*Perca fluviatilis*)^12^, three-spine sticklebacks (*Gasterosteus aculeatus*)^11^, and juvenile mosquitofish (*Gambusia holbrooki*)^10^. In the present study, we sought to expand upon the current understanding by investigating social facilitation in running waters. Interestingly, we found that the effect of group size on EA and TL seemed to vary with flow velocity, with minimal discernibility at the lowest velocity and becoming more prominent at higher velocities (Fig. 2 and Table 1). But why should flow velocity affect social facilitation? We propose three hypotheses explaining the observed behaviour: competition, stress reduction, and energy saving from coordinated swimming.

Competitive displacement may emerge among individuals seeking energetically favourable areas, typically characterized by lower flow velocities (e.g. near the flume walls^49^. Fish swimming alone tended to linger in these areas, resulting in reduced variability in their positional patterns compared to when swimming in groups (Fig. 3). Swimming with conspecifics introduces the potential for competition over these desirable locations, which may lead to greater spatial variability and increased exploration by each individual as they interchangeably displace each other while seeking the best spots. Flow velocity could mediate the intensity of such competition by exacerbating the necessity for seeking refuge in these areas at higher velocities. However, if competition were the sole factor at play, the impact on EA and TL would be less prominent. When examining medium and high velocities, we observe a substantial increase in average EA and TL, nearly doubling in two-fish groups compared to single-fish groups (Fig. 2). Given the near symmetry of the flume and flow-field conditions, even if both fish were to closely compete for a single favourable area and displace each other, it is unlikely that the overall covered area and trajectory would double as there would be plenty of other very favourable spots available nearby. Hence, it is improbable that competition alone accounted for the observed increase in EA and TL with group size at medium and high velocities.

The second hypothesis relates to the calming effect of social facilitation, which reduces stress levels in fish when in the presence of conspecifics^50^. Results from physiological analysis indicated that fish swimming in groups exhibited lower cortisol and oxidative stress levels in muscle tissue compared to solitary counterparts^30^. It is plausible that under low flow conditions, fish may experience reduced stress, thereby diminishing the significance of the presence of others (i.e. group effects such as social facilitation). Conversely, higher velocities might induce greater stress in fish, amplifying the impact of conspecific presence. Studies on rainbow trout (*Oncorhynchus mykiss*) and turbot (*Scophthalmus maximus*) have shown a relationship between stress and flow velocity, with higher stress responses and oxidative damage observed at higher flow velocities^51,52^.

The third explanation centres around the energetic benefits of swimming in groups^53^. Qualitative observations indicated a common tendency for fish to swim and school together, with some exceptions like solitary individuals near the downstream grid. Coordinated swimming (schooling) allows fish to take advantage of hydrodynamic features, resulting in energy conservation strategies^13–16,54,55^. As fish save energy in schools, they may allocate these reserves towards exploring their environment and exhibiting increased movement within the available arena when in groups, especially under challenging hydrodynamic conditions, namely at 20 and 35 cm/s.

To support this hypothesis, we assess the level of hydrodynamic challenge faced by the fish in our experiments by comparing the employed flow velocities with the so-called *Maximum Sustained Velocity* ($${U}_{ms}$$). $${U}_{ms}$$ identifies the crossover between the *sustained* and *prolonged swimming range*. The former indicates a range of velocities (lower than $${U}_{ms})$$ that fish can maintain for more than 200 min, using only aerobic energy reserves^56^. The latter instead identifies a range of velocities (higher than $${U}_{ms}$$) that are much more energetically demanding as they involve the use of anaerobic energy reserves, which eventually run out, hence leading fish to fatigue^56^. An initial estimate of $${U}_{ms}$$ can be retrieved using the Videler’s formula^28^, which in our case (i.e. fish with a body length of 5.14 cm) yields $${U}_{ms}=$$ 27 cm/s. A more robust reference for $${U}_{ms}$$ can be drawn from swimming performance curves of similar species sourced in databases^57^. For example, studies on the common dace (*Leuciscus leuciscus*), an ecologically similar fish species, indicate its capacity to sustain a speed of 5 body legths/s for durations ranging from 1 to 150 min, corresponding to a swimming speed of approximately 26 cm/s for the *Telestes muticellus* under study. This empirical evidence aligns closely with the prediction derived from Videler’s formula. These considerations imply that the lowest velocity utilised in our experiments (10 cm/s) was probably well within the sustained range and hence not energetically challenging for the fish. In contrast, the medium (20 cm/s) and highest investigated velocities (35 cm/s) are close to or greater than the estimated $${U}_{ms}$$, respectively, meaning they could belong to the more challenging prolonged range. Considering that the fish swam at the highest velocity for 10 min, it is reasonable to infer that they were far from fatigue, given that $${U}_{ms}$$ provides an order of magnitude for fish swimming continuously for 200 min (note that boundaries between different ranges are far from being sharp).

These considerations support the hypothesis that energetics might have played a role in modulating the effect of flow velocity on social facilitation observed in our study. Nevertheless, it remains challenging to discern whether the effects of flow velocity on social facilitation are attributable to a single mechanism or a combination of the three identified above. Furthermore, certain individuals and groups may be primarily influenced by one mechanism, while others may experience a greater impact from another due to the substantial intraspecific variability observed in wild animals^58^. Nonetheless, these findings fulfil a dual purpose by highlighting the critical role of flow velocity in behavioural research on rheophilic species^59–62^ and by emphasising the importance of integrating group behaviour in the field of ecohydraulics.

Overall, these findings have practical applications for conservation management purposes. Despite the extensive construction of fish passages to mitigate freshwater migratory fish decline, the efficacy of these structures is debated, as numerous structures suffer from low passage efficiency^63,64^. An effective exploratory behaviour is crucial for fish to search and locate entrances of fishways at river barriers^63–65^, and while extensive research has focused on enhancing the attractiveness of such entrances for individual fish^64,66,67^, very little is known about the case of shoaling fish^17,68^. Emerging experimental evidence, such as studies on gudgeons (*Gobio gobio*)^69^ and barbels (*Barbus barbus*)^70^, suggests that aggregation can lead to increased passage efficiency. In pacific salmon (genera *Oncorhynchus* and *Salmo*), the presence of conspecifics is suggested to facilitate both large-scale navigation^71^ and higher fish passage efficiency^72^. In a telemetry study, Okasaki et al. found that Chinook salmon (*Oncorhynchus tshawytscha*) located fishway entrances more quickly and required fewer attempts to pass when in high group densities^72^. These findings highlight the importance of the present work and similar future endeavours for practical conservation purposes. Additionally, gaining insights into the spatial utilisation of fish schools, both horizontally and vertically, in confined environments can provide relevant evidence for the efficient design of fish passages, resulting in optimised configurations that align with fish spatial preferences, particularly under challenging flow conditions^73^.

## Conclusions

This study offers insights into the interplay between flow velocity and social facilitation on fish exploration and swimming activity. We found that social facilitation occurs even with as few as two fish, but its effects vary depending on flow velocity. This means that caution should be exerted when interpreting findings from experiments conducted in aquariums and tanks with stagnant waters, as they may not entirely capture the effects of social facilitation on fish behaviour, particularly for rheophilic species. Since river habitats often exhibit diverse flow conditions, neglecting hydrodynamics could lead to unrepresentative or biased outcomes. Conversely, a comprehensive understanding of collective behaviour becomes imperative when investigating the behaviour of gregarious species inhabiting flowing waters. In essence, this study underscores the necessity of accounting for the intricate relationship between hydrodynamics and social interactions to gain a more holistic perspective on fish behaviour.

### Supplementary Information

Supplementary Information.

## Data Availability

The data underlying the findings of this study are available upon reasonable request. Researchers interested in accessing the data can contact the corresponding authors.
