# Supplementary material for: The interplay of group size and flow velocity modulates fish exploratory behaviour
Source: Sci Rep. 2024 Jun 8;14:13186. doi: 10.1038/s41598-024-63975-z (PMC11162439; doi:10.1038/s41598-024-63975-z)
Supplement: Supplementary file 1 — Supplementary Information. [file 41598_2024_63975_MOESM1_ESM.docx]

Supplementary material


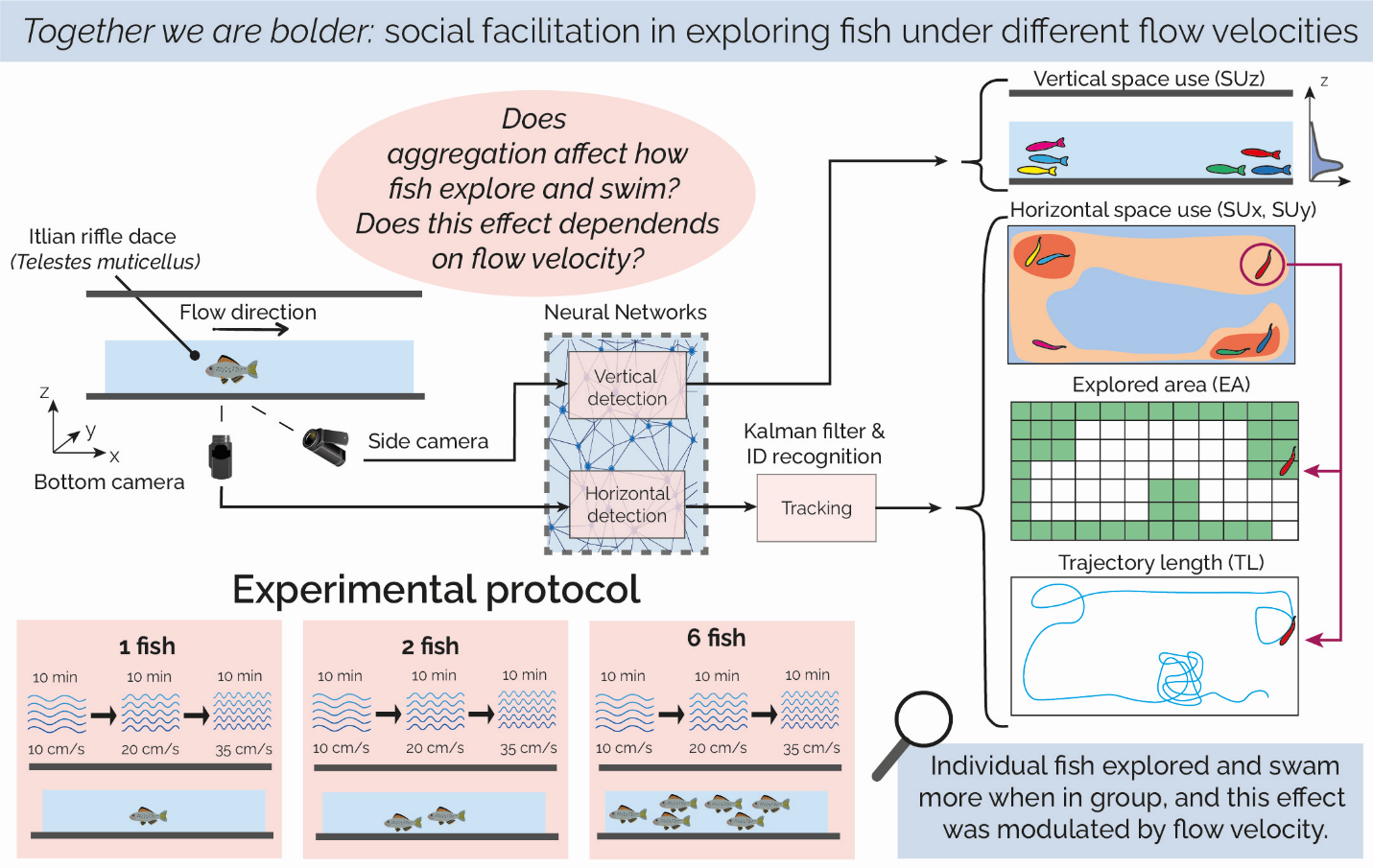


Figure S1: Graphical summary of the study

# Detection

## Training

We trained two YOLOv4 Convolutional Neural Networks (CNNs) using the Darknet framework (1). The training dataset consisted of approximately 10,000 images from each camera view (bottom and side), with resolutions of 1920 x 1080 pixels. To optimize training efficiency, the images were cropped into two parts and used for training. Additionally, images without fish were included to maximize detection performance. The parameters used to train the CNNs are provided in Table S1.

Table S1: Training parameters used for the CNNs, both for the bottom and side videos

| Parameter |  |
| --- | --- |
| Architecture | YOLOv4 |
| Number of input images | 10 000 |
| Input image resolution* | 1020x1080 |
| Number of iterations | 6000 |
| Batch size | 64 |
| Subdivisions | 64 |
| Training\Testing ratio | 85/15 |
| Resolution after down-sizing | 384x384 |

*images were divided in half, so the number of input images for the network doubled

## Testing CNN performances

When evaluating the performance of neural networks for object detection, various parameters can be used. One commonly employed parameter is the F1 score (2), which represents the harmonic mean of precision and recall:

$$Precision=\frac{TP}{TP+FP}$$

$$Recall=\frac{TP}{TP+FN}$$

$$F1=2\cdot\frac{Precision*Recall}{TP+FPPrecision+Recall}$$

Where $TP$, $FP$*,* $FN$ are the number of true positive, false positive and false negative, respectively. Another metric commonly used to evaluate detection performance is the mean average precision value (mAP) (3). It is calculated by averaging the average precision (AP) values for each class across different intersection-over-union (IoU) thresholds. The AP represents the area under the precision-recall curve for a specific class. The IoU threshold determines the required overlap between the predicted bounding box and the ground truth bounding box for a detection to be considered correct. A higher mAP50 value indicates a more accurate alignment of the network with the mark around the fish's head.

Table S2 and Table S3 present an overview of how these performance indicators vary with increasing iteration numbers for the bottom and side CNN, respectively. After two training sessions with 6,000 iterations each, the indicators for the bottom CNN were as follows: precision, recall, mAP50, and F1 score were all 96%. For the side CNN, the precision was 90%, recall was 93%, mAP50 was 98%, and the F1 score was 92%.

To filter the results of the detection process, a confidence level threshold of 0.3 was applied (4). In the case of false positives - where more detections were obtained than the actual number of fish present in the flume - detections with higher confidence levels were prioritized and selected.

Table S2: Bottom CNN performances with training iteration number

| Iterations | 0 | 1000 | 2000 | 3000 | 4000 | 5000 | 6000 |
| --- | --- | --- | --- | --- | --- | --- | --- |
| Detections | 2653347 | 10841 | 7213 | 7774 | 7189 | 7943 | 8055 |
| Ground-truth | 3574 | 3574 | 3574 | 3574 | 3574 | 3574 | 3574 |
| mAP0.50 | 0 | 90.78 | 93.2 | 94.21 | 94.48 | 95.76 | 95.89 |
| Precision0.25 | 0 | 0.93 | 0.95 | 0.96 | 0.95 | 0.96 | 0.96 |
| Recall0.25 | 0 | 0.90 | 0.95 | 0.95 | 0.95 | 0.95 | 0.96 |
| F1 | 0 | 0.91 | 0.95 | 0.95 | 0.95 | 0.96 | 0.96 |
| TP (True Positive) | 0 | 3207 | 3384 | 3383 | 3396 | 3413 | 3415 |
| FP (False Positive) | 2541117 | 254 | 174 | 144 | 174 | 143 | 141 |
| FN (False Negative) | 3574 | 367 | 190 | 191 | 178 | 161 | 159 |
| Average IoU | 0 | 67.48 | 73.34 | 73.85 | 74.12 | 74.83 | 75.24 |

Table S3: Side CNN performances with training iteration number

| Iterations | 0 | 1000 | 2000 | 3000 | 4000 | 5000 | 6000 |
| --- | --- | --- | --- | --- | --- | --- | --- |
| Detections | 12530 | 12530 | 5890 | 6377 | 5535 | 5075 | 4882 |
| Ground-truth | 2963 | 2963 | 2963 | 2963 | 2963 | 2963 | 2963 |
| mAP0.50 | 0 | 90.62 | 96.66 | 96.13 | 97.38 | 97.48 | 97.45 |
| Precision0.25 | 0 | 0.73 | 0.87 | 0.87 | 0.88 | 0.90 | 0.90 |
| Recall0.25 | 0 | 0.80 | 0.90 | 0.91 | 0.92 | 0.94 | 0.93 |
| F1 | 0 | 0.76 | 0.88 | 0.89 | 0.9 | 0.92 | 0.92 |
| TP (True Positive) | 0 | 2375 | 2674 | 2696 | 2730 | 2774 | 2769 |
| FP (False Positive) | 899534 | 899 | 411 | 389 | 376 | 314 | 317 |
| FN (False Negative) | 2963 | 588 | 289 | 267 | 233 | 189 | 194 |
| Average IoU | 0 | 52.74 | 68.04 | 68.69 | 70.27 | 73.52 | 73.48 |

## Detections along the vertical coordinate

In the side videos capturing six-fish groups, a high occurrence of fish swimming side-by-side at the bottom of the flume was observed. This led to numerous complete occlusions, where fish positioned closer to the camera would obstruct those swimming next to them. To assess the relevance of this issue, the number of missing detections was quantified. For each side video, the missing detections ($MD$) were calculated as:

$${MD}_{f}=1-\frac{N_{CNN det}}{f\cdot N_{frames}}$$

Where $N_{CNN det}$ is the number of CNN detections in the trial, $f$ is the group size (i.e. number of fish present in the flume), and $N_{frames}$ is the number of frames in the video trial. On average, ${MD}_{1}$ (i.e. ${MD}_{f}$ with $f$ =1, which is the case of single fish) was approximately 16%. This value was attributed to fish swimming in areas with low contrast with the background, such as the upstream honeycomb, or when they were at rest or impinged against the downstream grid. In the case of two-fish groups, the average ${MD}_{2}$ was around 24%, while for six-fish groups, ${MD}_{6}$ escalated to 45%. The increasing number of ${MD}_{f}$ with group size ($f$) can be attributed to the numerous occlusions arising from fish swimming side-by-side in the side camera view. Although the rate of ${MD}_{1}$ for single fish was deemed acceptable for input into our tracking algorithm, the elevated occlusion rate for six-fish groups hindered the efficacy of the custom-made algorithm described below. Vertical space use analysis was therefore performed from CNN detections without additional filtering. It is worth emphasizing that the elevated occurrence of occlusions primarily took place in the lower section of the flume. This means that the depth (i.e., distance from the flume bottom) of fish was slightly overestimated for two-fish groups and significantly overestimated for six-fish groups. This overestimation was not considered a limitation, as it actually provided a conservative result aligning with the assumption made for our horizontal 2D analysis presented in the “Result” section in the main paper (“3.1 Explored area and trajectory length (EA and TL)”).

# Tracking

A Kalman filter (5) was implemented in order to retrieve a filtered trajectory of the fish from the noisy and/or missing detections (i.e. measurements). The filter applied uses a 2D (*x,y*) model with constant velocity.

The state vector describes the state of the fish at a certain time-step *t*:

$$\vec{x}_{t}=\left[ \begin{aligned} x \\ y \\ \begin{aligned} \dot{x} \\ \dot{y} \end{aligned} \end{aligned} \right]$$

Which comprises the position coordinates ($x, y$) and their velocity ($\dot{x},\dot{y}$). After every time step ($\Delta t$), the model produces a predicted state $x^{p}$ and an estimated state $x^{e}$ based on actual measurements ($z_{t}$) of the object. Being $x_{CNN,t}$ and $y_{CNN,t}$ the detections from the CNN at the time step $t$, the actual measurement ($z_{t}$) can be calculated as:

$$z_{t}={[x_{CNN,t},y_{CNN,t}, \frac{x_{CNN,t}-x_{CNN,t-1}}{\Delta t},\frac{y_{CNN,t}-y_{CNN,t-1}}{\Delta t}]}^{T}$$

## Initial state

The initial estimated state will start from the initial measure:

$$x_{0}^{e}=z_{0}=\left[ \begin{aligned} x_{CNN,0} \\ y_{CNN,0} \\ \begin{aligned} 0 \\ 0 \end{aligned} \end{aligned} \right]$$

In order to begin the prediction of the next state, the estimate uncertainty matrix (or covariance matrix) needs to be computed. This matrix represents the covariance between the error in the estimated state and the error in the measurement of the state, and provides an indication on the accuracy of the state estimate. Assuming no correlation between estimation errors on *x* and *y*, and position and velocity, the covariance matrix becomes a diagonal matrix. The initial covariance matrix is:

$$P_{0}=\left[ \begin{matrix} p_{x} & 0 & \begin{matrix} 0 & 0 \end{matrix} \\ 0 & p_{y} & \begin{matrix} 0 & 0 \end{matrix} \\ \begin{aligned} 0 \\ 0 \end{aligned} & \begin{aligned} 0 \\ 0 \end{aligned} & \begin{aligned} \begin{matrix} p_{\dot{x}} & 0 \end{matrix} \\ \begin{matrix} 0 & p_{\dot{y}} \end{matrix} \end{aligned} \end{matrix} \right]$$

## Prediction

The current predicted state is based on the previously estimated state:

$$x_{t}^{p}=Ax_{t-1}^{e}$$

Where *A* is the dynamic matrix (or state transition matrix):

$$A=\left[ \begin{matrix} 1 & 0 & \begin{matrix} \Delta t & 0 \end{matrix} \\ 0 & 1 & \begin{matrix} 0 & \Delta t \end{matrix} \\ \begin{aligned} 0 \\ 0 \end{aligned} & \begin{aligned} 0 \\ 0 \end{aligned} & \begin{aligned} \begin{matrix} 1 & 0 \end{matrix} \\ \begin{matrix} 0 & 1 \end{matrix} \end{aligned} \end{matrix} \right]$$

Also, the predicted covariance matrix is uopdated based on the previous step:

$$P_{t}=AP_{t-1}A^{T}+Q$$

With *Q* being the process noise covariance matrix, which represents the uncertainty of the process and measurement noises:

$$Q=\sigma_{a}^{2}G\cdot G^{T}$$

Being $\sigma_{a}$ the standard deviation on the acceleration and $G:$

$$G=\left[ \begin{aligned} \frac{1}{2}{\Delta t}^{2} \\ \frac{1}{2}{\Delta t}^{2} \\ \begin{aligned} \Delta t \\ \Delta t \end{aligned} \end{aligned} \right]$$

## Estimation

After the prediction, the estimation is updated with the actual measurement $z_{t}$.

$$x_{t}^{e}=x_{t}^{p}+K(Mcz_{t}-Hx_{t}^{p})$$

Where *H* is the measuring matrix:

$$H=\left[ \begin{matrix} 1 & 0 & \begin{matrix} 0 & 0 \end{matrix} \\ 0 & 1 & \begin{matrix} 0 & 0 \end{matrix} \\ \begin{aligned} 0 \\ 0 \end{aligned} & \begin{aligned} 0 \\ 0 \end{aligned} & \begin{aligned} \begin{matrix} 1 & 0 \end{matrix} \\ \begin{matrix} 0 & 1 \end{matrix} \end{aligned} \end{matrix} \right]$$

And *K* is the Kalman gain:

$$K_{t}=\frac{P_{t-1}H^{T}}{HP_{t-1}H^{T}+R}$$

With *R* being the noise covariance matrix:

$$R=\left[ \begin{matrix} \sigma_{x} & 0 & \begin{matrix} 0 & 0 \end{matrix} \\ 0 & \sigma_{y} & \begin{matrix} 0 & 0 \end{matrix} \\ \begin{aligned} 0 \\ 0 \end{aligned} & \begin{aligned} 0 \\ 0 \end{aligned} & \begin{aligned} \begin{matrix} \sigma_{\dot{x}} & 0 \end{matrix} \\ \begin{matrix} 0 & \sigma_{\dot{y}} \end{matrix} \end{aligned} \end{matrix} \right]$$

The covariance matrix is also then updated:

$$P_{t-1}=\left( I-K_{t}H \right)*P_{t-1}$$

Note that the Kalman gain *K* indicates whether the model should trust the measurement ($K\to1$) or the prediction ($K\to0$). With this method, at the time steps where there is a missing detection, the Kalman gain will be zero, and the state of the fish will be estimated from previous states (assuming constant velocity).


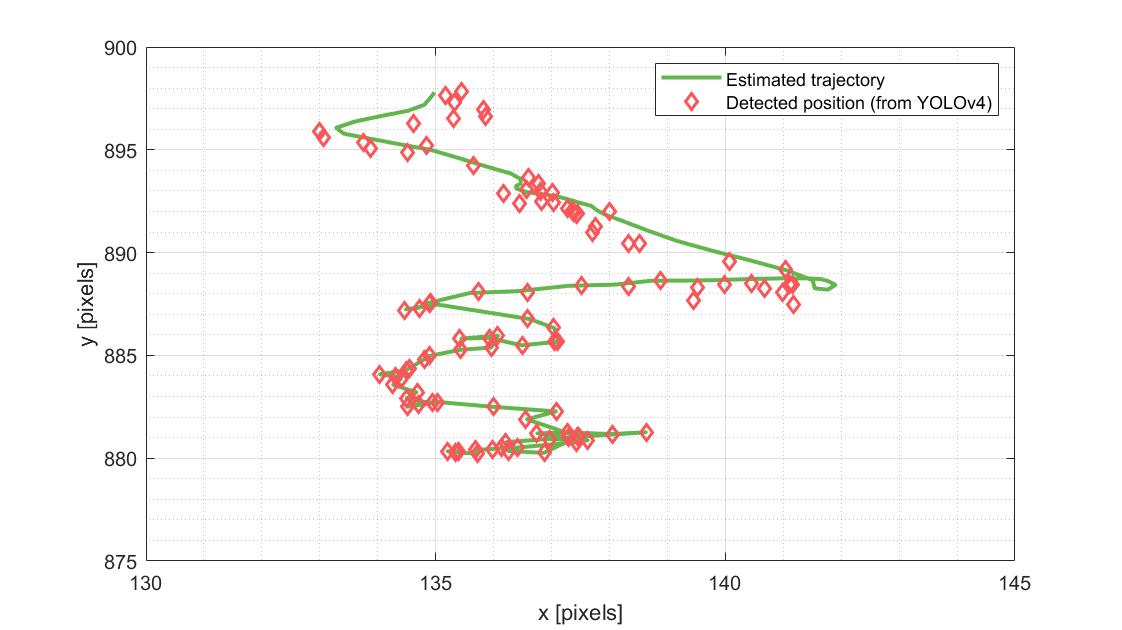


Figure S2: Example of observed and estimated position for single fish

## Velocity dumper

By using a constant velocity model, the filter estimates an undetected object by following a linear trajectory. As a result, for time periods in which the object is not detected, the estimated position keeps moving at a constant velocity, and when the non-detection interval is long, the object can also move out of the boundaries. In order to solve this issue, a condition on the prediction was added if the position was not measured:

$$if z_{t}=NaN \to x_{t}^{p}=D\cdot Ax_{t-1}^{e}$$

$$D=\left[ \begin{matrix} 1 & 0 & \begin{matrix} 0 & 0 \end{matrix} \\ 0 & 1 & \begin{matrix} 0 & 0 \end{matrix} \\ \begin{aligned} 0 \\ 0 \end{aligned} & \begin{aligned} 0 \\ 0 \end{aligned} & \begin{aligned} \begin{matrix} d & 0 \end{matrix} \\ \begin{matrix} 0 & d \end{matrix} \end{aligned} \end{matrix} \right]$$

Where *d* ($0\leq d\leq1$) is a dumper that reduces the velocity from the previous time step.

## Identity recognition

In the case of single fish, the Kalman filter can be applied to the measurement to retrieve a filtered trajectory. For multiple fish, however, it is necessary to relate the object of a new time step to the previous one (see Figure S3). This was done by assigning a probability to all possible configurations of the fish identities between consecutive time steps (permutations). Only two combinations (*N*=2) are possible in the case of two fish. In the case of 6 fish, however, there are 720 possible permutations (*N*=720).

For each configuration, the sum of the norms of the acceleration vectors is calculated.

$$c_{i,t}=\sum_{k}^{Nfish} \left| \vec{a}_{k,t} \right|$$

$$\vec{a}_{k,t}=\frac{\vec{v}_{k,t}-\vec{v}_{k,t-1}}{\Delta t}$$

$$\vec{v}_{k,t}=\frac{z_{k,t}-{L\cdot x}_{k,t-1}^{e}}{\Delta t}$$

$$\vec{v}_{k,t-1}={V\cdot x}_{k,t-1}^{e}$$

Where *L* and *V* are matrixes that extract position and velocity from the state vector, respectively:

L=$\left[ \begin{matrix} 1 & 0 & \begin{matrix} 0 & 0 \end{matrix} \\ 0 & 1 & \begin{matrix} 0 & 0 \end{matrix} \end{matrix} \right]$

V=$\left[ \begin{matrix} 0 & 0 & \begin{matrix} 1 & 0 \end{matrix} \\ 0 & 0 & \begin{matrix} 0 & 1 \end{matrix} \end{matrix} \right]$

The probability of each configuration *q* is then calculated as:

$$q_{i,t}=1-\frac{c_{i,t}}{\sum_{j=1}^{N} c_{j,t}}$$

$$\left\{ \begin{aligned} if Nfish=2 \to N=2 \\ if Nfish=6 \to N=720 \end{aligned} \right.$$

Identity was therefore assigned by selecting the configuration with the highest probability among the *N* possible permutations:

$$c_{t}={max}_{i=1..N}(q_{i,t})$$

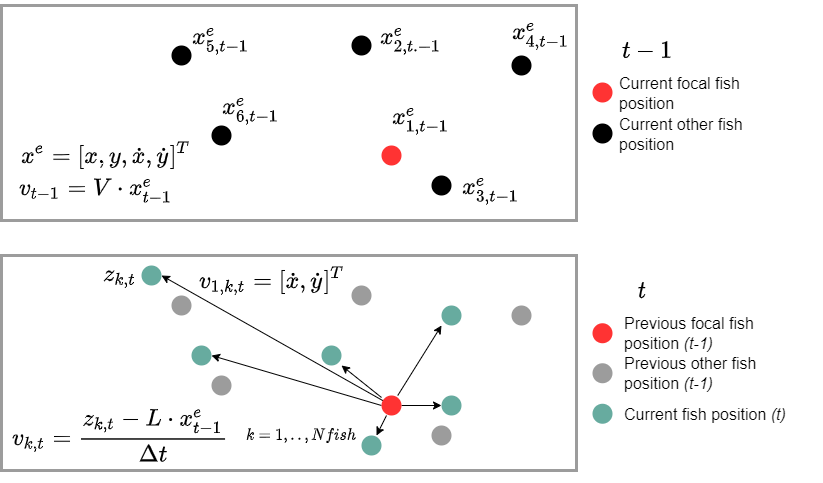


Figure S3: State and velocities for two consecutive time steps


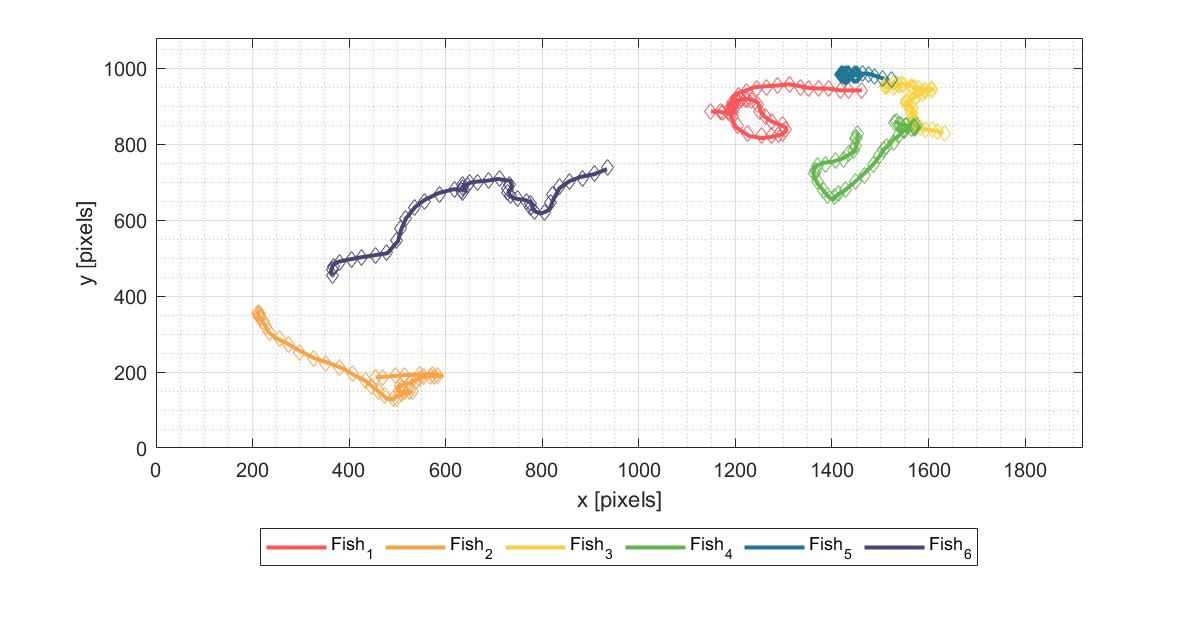


Figure S4: Example of observation and estimated positions for six fish

## Validation

The tracking performance of videos captured by the bottom camera was assessed by evaluating the accuracy of the identity algorithm. The primary objective was to examine the assignment of identities during occlusions, which occurred when two or more fish crossed paths. The algorithm's performance was visually verified to determine if the assigned identities were correct or if there were instances of swapping. To minimize the occurrence of identity swaps during occlusions, filter parameters were optimized using a trial-and-error approach. Visual confirmation was conducted on a randomly selected two-minute subset of videos from three velocity treatments, resulting in a total of 50 occlusions. The optimized filter parameters yielded an identity accuracy of 87% for the analyzed occlusions.

# Randomisation

The study comprised a total of 100 fish: 20 trials for single fish (i.e., 20 fish), 10 trials for two-fish groups (i.e., 20 fish), and 10 trials for six-fish groups (i.e., 60 fish), with each fish tested only once. Randomisation was obtained by employing a block sequence comprising two trials with single fish (labelled as “A”), one trial with two fish (“B”), and one trial with six fish (“C”). Each unique combination of these trials within a block was assigned a number from 1 to 15 (e.g., 1 = “AABC”, 2 = “AACB”, 3 = “ABAC”, etc.). Subsequently, we utilised the '*rand*' command in MATLAB (6) to generate a sequence of 10 random numbers ranging from one to 15. This process yielded a random sequence of 10 blocks, each consisting of two single-fish trials, one two-fish trial, and one six-fish trial.

# Bottom-filtered horizontal analysis

In order to determine whether results from the horizontal analysis were biased by fish movement in the water column, we conducted an additional bottom-filtered analysis. This analysis was performed by removing frames in which at least one fish in the group was detected higher than 5 cm from the bottom. Bottom-filtered explored area (EA*), trajectory length (TL*), and horizontal space use ($P_{H}^{*}(x,y)$), were computed for all the trials. As the trajectory length accumulates over the number of frames, TL* was adjusted by considering the number of non-filtered frames as follows:

$${TL}^{*}=TL\cdot\frac{F_{tot}}{F_{bottom}}$$

Where $F_{tot}$ represented the total number of frames in each trial, and $F_{bottom}$ the number of frames in which no fish was detected above 5 cm.

Upon visual comparison of bottom filtered EA* and TL* (Figure S5) with the unfiltered data (Figure 1 in the “Results” section), it can be seen that the main trend remained consistent. Similar to the unfiltered analysis of the entire water volume, group size did not impact EA* and TL* at the lowest velocity (10 cm/s). However, EA* demonstrated an increase with larger group sizes at both medium and high velocities (20 and 35 cm/s, respectively), replicating the findings observed in the unsfiltered analysis on EA. Similarly, the trend of TL* with group size was similar to that observed in the entire volume analysis (TL). Specifically, at the medium velocity, six-fish and two-fish groups exhibited comparable TL* values, significantly higher than those of single fish. Conversely, at the highest velocity, an increase in TL* was observed with larger group sizes, perfectly mirroring the results from the unfiltered analysis. Comparing differences in means, it can be seen that the variation in EA* stay below 7.5% (|EA-EA*|, Table S4), while for TL is below 1.5 cm (|TL-TL*|, Table S4). Also regarding horizontal space use ($P_{H}^{*}\left( x,y \right),$ Figure S6), the distributions are highly similar to what observed in the entire volume ($P_{H}(x,y)$, Figure 2 in the “Results” section). Variations in medians positions are less than 2 cm for the longitudinal position ($\left| P_{H}^{*}\left( x \right)-P_{H}\left( x \right) \right|$, Table S4) 1.1 cm for the lateral one ($\left| P_{H}^{*}\left( y \right)-P_{H}\left( y \right) \right|$, Table S4).


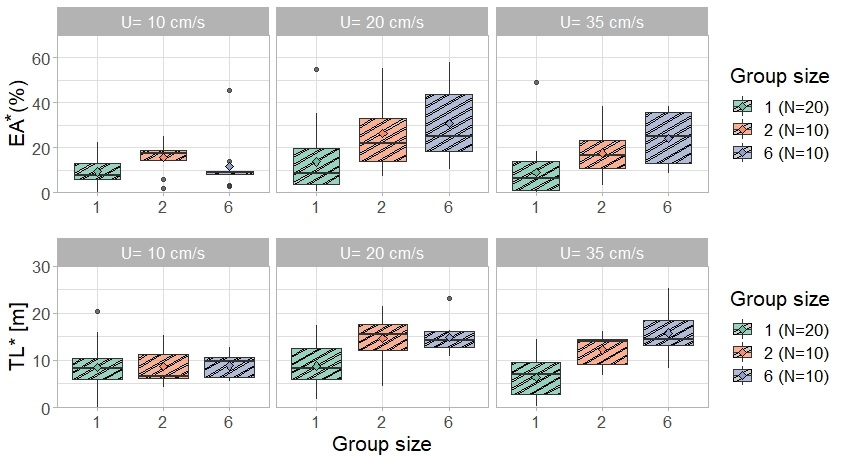


Figure S5: Bottom-filtered explored area (EA*, top) and trajectory length (TL*, bottom) by each fish in the horizontal plane. Filtering was implemented by considering only frames in which no fish was detected above 5cm from the flume bottom. Values are averaged for the group and the rhombi represent the means. TL* is adjusted for the number of non-filtered frames (TL*=TL · F_tot_ /F_bottom_, with F_tot_ being the total number of frames in each trial and F_bottom_ the number of frames in which no fish was detected above 5cm in the vertical coordinate).


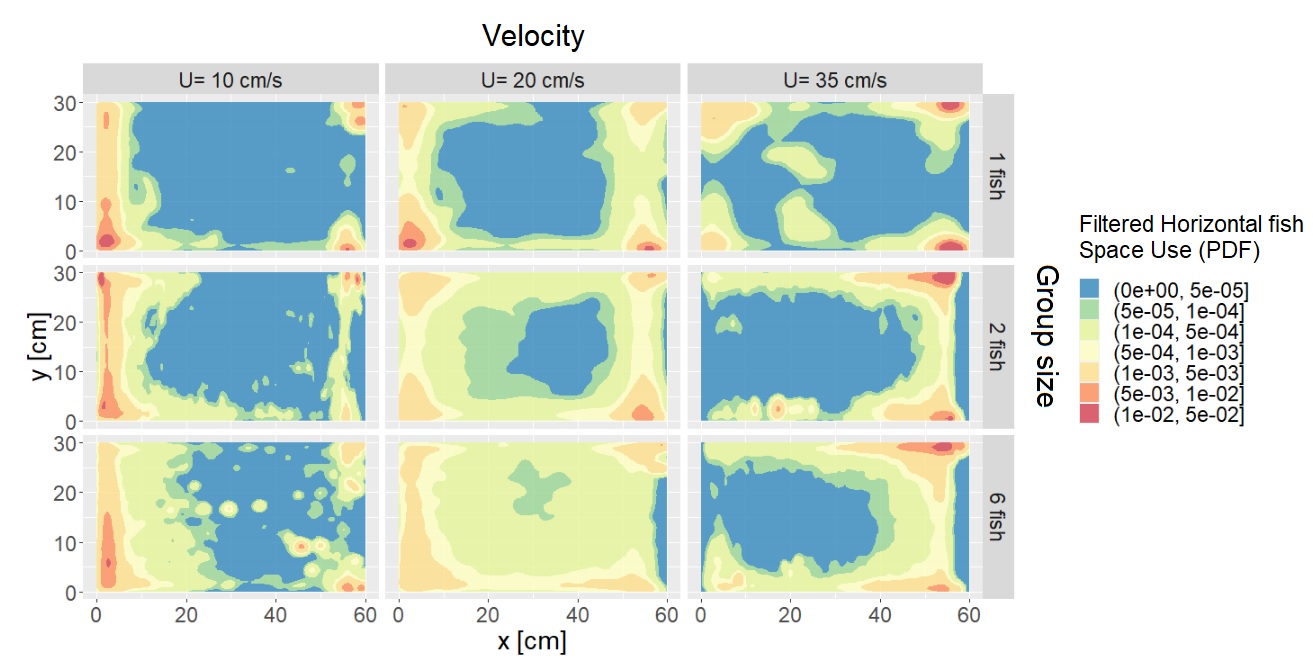


Figure S6: Bottom-filtered orizontal probability density (P_H_*) illustrating fish distribution at various flow velocities (10, 20, and 35 cm/s) and group sizes (1, 2, and 6). Filtering was performed by disregarding frames in which at least one fish was detected above 5cm from the flume bottom. Water is flowing from left to right.

Table S4: Mean explored area and trajectory length for unfiltered analysis (EA, and TL) and for bottom-filtered analysis (EA*, and TL*), and median values of longitudinal and lateral position ($\tilde{P}_{H}\left( x \right)$ and $\tilde{P}_{H}\left( y \right)$ for unfiltered data, and $\tilde{P}_{H}^{*}\left( x \right)$and $\tilde{P}_{H}^{*}\left( y \right)$ from bottom filtered analysis). Filtering was performed by removing frames in which at least one fish was detected above 5 cm in the water column.

|  | Mean explored area (%) | | Mean trajectory length [m] | | Median longitudinal position [cm] | | Median lateral position [cm] | |
| --- | --- | --- | --- | --- | --- | --- | --- | --- |
|  | $\bar{EA}$ | $\bar{{EA}^{*}}$ | $\bar{TL}$ | $\bar{{TL}^{*}}$ | $\tilde{P}_{H}\left( x \right)$ | ${\tilde{P}_{H}}^{*}\left( x \right)$ | $\tilde{P}_{H}\left( y \right)$ | $\tilde{P}_{H}^{*}\left( y \right)$ |
| 1 fish, 10 cm/s | 9.9 | 9.2 | 8.3 | 9.8 | 2.57 | 2.67 | 9.75 | 9.19 |
| 1 fish, 20 cm/s | 14.0 | 13.6 | 9.4 | 10.2 | 4.55 | 3.87 | 5.47 | 4.83 |
| 1 fish, 35 cm/s | 8.8 | 8.8 | 6.1 | 6.5 | 55.3 | 55.3 | 23.7 | 23.7 |
| 2 fish, 10 cm/s | 20.1 | 15.6 | 12.0 | 12.4 | 3.72 | 3.54 | 15.6 | 14.5 |
| 2 fish, 20 cm/s | 27.1 | 26.4 | 14.4 | 14.5 | 35.0 | 33.6 | 8.88 | 8.19 |
| 2 fish, 35 cm/s | 17.6 | 17.5 | 11.2 | 12.2 | 52.3 | 52.4 | 28.0 | 27.8 |
| 6 fish, 10 cm/s | 19.1 | 11.7 | 11.1 | 10.4 | 4.09 | 3.82 | 9.08 | 9.08 |
| 6 fish, 20 cm/s | 38.3 | 30.8 | 16.5 | 16.3 | 16.9 | 18.9 | 11.4 | 10.8 |
| 6 fish, 35 cm/s | 26.1 | 24.0 | 14.7 | 14.6 | 49.6 | 49.8 | 26.7 | 26.7 |

# Hydrodynamic characterisation of the flume

Table S5: Reynolds and Froude numbers for the three velocitiy treatments utilised in the experimental protocol

| Velocity treatment | Re | Fr |
| --- | --- | --- |
| *U* = 10 cm/s | 5.13·10^4^ | 0.08 |
| *U* = 20 cm/s | 10.2·10^4^ | 0.16 |
| *U* = 35 cm/s | 17.9·10^4^ | 0.29 |

## Computational Fluid Dynamics

Computational Fluid Dynamics (CFD) simulations, run using ANSYS Fluent software (Canonsburg, Pennsylvania, USA), were used to provide a detailed characterization of flow field variations within the test section of the flume. This allowed to accurately represent flow velocities experienced by fish in near-wall regions, namely the bottom and side walls of the flume, where the presence of a boundary layer typically results in lower flow velocities than elsewhere. The computational domain, measuring 60 cm x 30 cm x 15 cm (length x width x depth), was discretized into finite elements using a structured grid composed of hexahedral cells.

Cells of size 5 mm were used at the center of the computational domain whereas 20 inflation layers, with smooth transition inflation option, consisting of cells with progressively increasing height were used to accurately capture the flow velocity in the boundary layer region. This resulted in a refined mesh near walls and a coarser mesh farther away, effectively optimizing computational efficiency while maintaining mesh-independent outputs. The entire domain was subdivided into 495040 nodes and 478018 elements. Regarding mesh quality, it was ensured that the aspect ratio and the orthogonal quality metric values were close to unity, and the value of skewness was less than 0.2. Steady-state simulations were run whereby pressure-based solver was employed, as typically used for incompressible flows. Modeled flow velocities ($\bar{u}$, $\bar{v}$, $\bar{w}$), where $\bar{u}$, $\bar{v}$, and $\bar{w}$ are the longitudinal, lateral, and vertical velocity components (overbar denotes time-averaging), were numerically computed at each node of the structured mesh. To obtain an accurate numerical solution, convergence criterion was effectively monitored. In all solutions, the residual errors decreased to values of the order of 10^-6^ or lower. Moreover, independent of the flow velocity treatment, no mass imbalances were obtained since the mass flow at the inlet and the outlet were similar.

Using Laser Doppler Anemometry (LDA) by Dantec Dynamics, direct velocity fluctuation measurements were taken 5 cm from the upstream grid. The turbulent kinetic energy (*k*) was then calculated from these LDA measurements. Following (7), a *k*-epsilon (*k*-*ε*) turbulence closure model was used for all CFD simulations which provided reliable results and was computationally efficient. Turbulent dissipation rate (*ε*) was estimated as *ε* = *U^3^/l_ms_*, where *U* is the average cross-sectional flow velocity and *l_ms_* is the characteristic length (based on upstream grid mesh size of 6 mm).

A mass flow inlet boundary condition was used at the inlet as the volume discharge entering the flow domain was known. The free surface was modelled as a symmetry plane, while the outlet was assigned with an outflow boundary condition. Both the bottom and side walls were specified as hydrodynamically smooth walls with a no-slip condition.

Figure S8 displays mean longitudinal flow velocity ($\bar{u}$) distribution for four lateral cross sections located at 5, 10, 30, and 50 cm from the upstream grid (Figure S7), for treatment with a mean cross-sectional flow velocity (*U*) of 10 cm/s. The velocity distribution was mostly uniform throughout the various cross sections, except for areas close to the wall, where the presence of a boundary layer resulted in lower $\bar{u}$ values. The thickness of the boundary layer increased with increasing downstream distance from the upstream grid (Figure S8). For the remaining two velocity treatments (i.e., *U* values of 20 and 35 cm/s), similar results were obtained (not shown here).


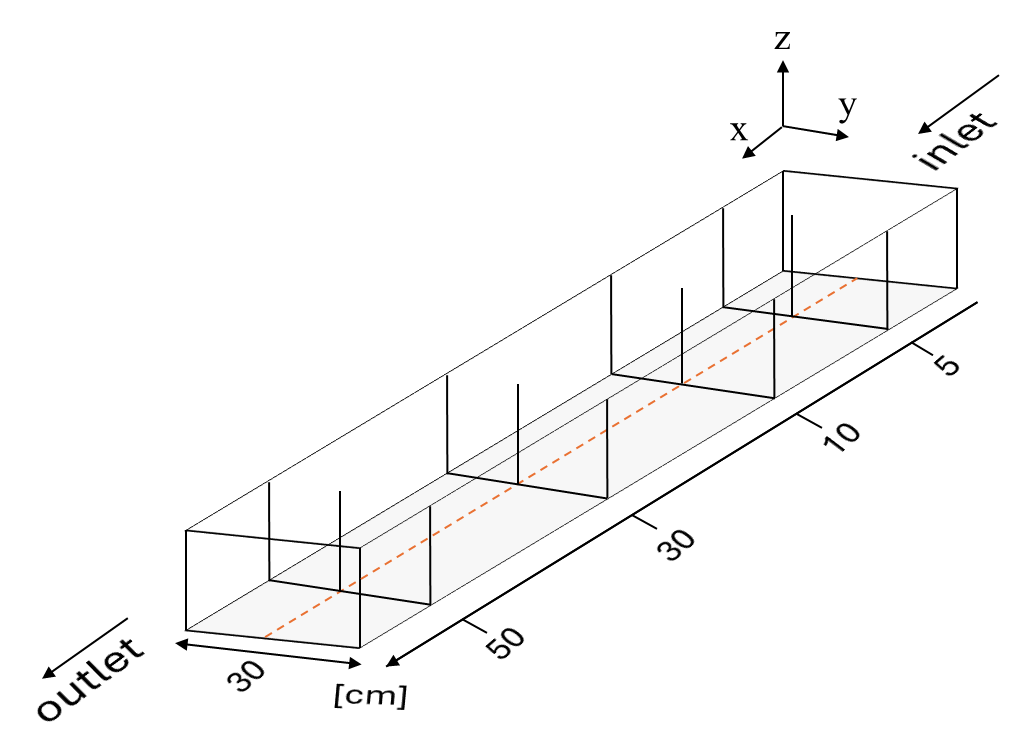


Figure S7: A schematic figure showing the locations of the four cross sections within the test section of the open channel flume. The longitudinal flow velocity measurements were taken, using LDA, at the center of each cross section along the vertical black line.


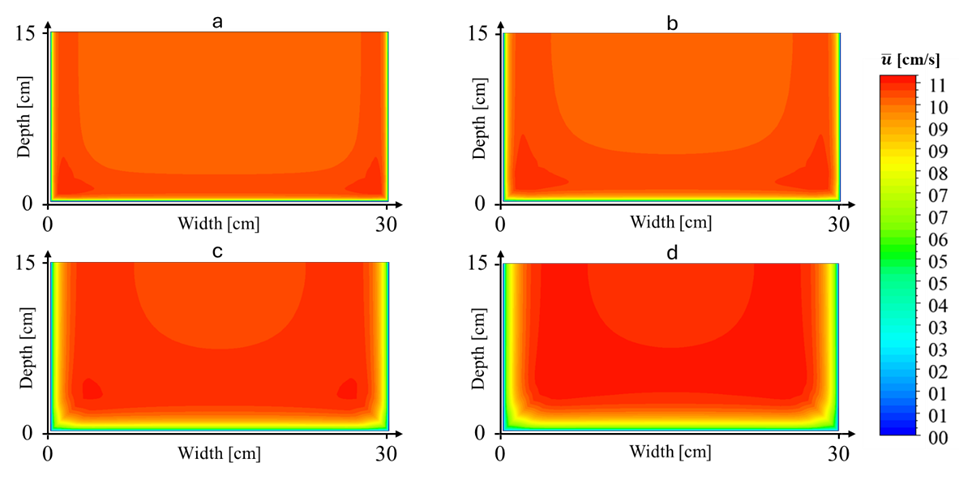


Figure S8: Lateral cross sections with longitudinal flow velocity magnitude obtained using CFD simulations for treatment with a mean flow velocity of 10 cm/s. The cross sections are located at a distance of (a) 5 cm, (b) 10 cm, (c) 30 cm, and (d) 50 cm from the upstream grid.

## Laser Doppler Anemometer

Single-point longitudinal flow velocities (*u*) were measured using Laser Doppler Anemometer (LDA). For all flow velocity treatments, measurements were taken at four cross-sections located at a distance of 5, 10, 30, and 50 cm from the upstream grid (Figure S1). Within each cross-section, measurements were obtained in the center along a vertical column at ten points, ranging from 0.5 to 15 cm with a vertical spacing of 1.5 cm. At each point, 10,000 measurements were taken at an average sampling frequency of about 100 Hz. As an example, Figure S9 shows the validation of CFD-simulated and LDA measured mean flow velocity for the treatment with *U* of 10 cm/s.

Mean absolute error (MAE) and root mean squared error (RMSE) were computed as statistical performance metrics to check the agreement between CFD-simulated and LDA measured mean flow velocities. The higher values of MAE and RMSE at 5 cm is due to the effect of upstream grid generated turbulence which diminishes with increasing downstream distance from the upstream grid. Overall, the results show that the agreement is very good, hence providing confidence that the simulated flow fields accurately represent experimental conditions. The numerically simulated and empirically measured flow velocity data, being independent and adhering to the criteria of normal distribution and homogeneity of variances, were compared using a two-sample t-test. At all cross sections and for all flow velocity treatments, no significant differences were found between the two velocity profiles (p-value > 0.05).


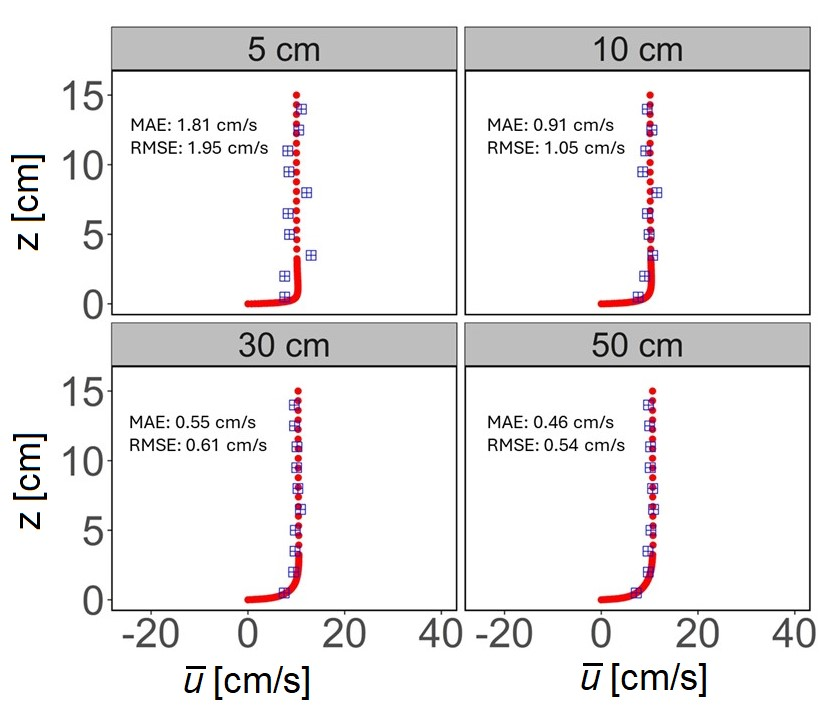


Figure S9:Comparison between mean longitudinal velocity profiles obtained from Computational Fluid Dynamics (CFD) simulations (red dots) and from Laser Doppler Anemometry (LDA) measurements (blue squares with plus symbol) for a treatment with bulk flow velocity of 10 cm/s. The plotted mean velocity profiles were obtained at the mid cross section of the flume at 5, 10, 30, and 50 cm from the upstream grid.

# Supplementary References

1. Jiang P, Ergu D, Liu F, Cai Y, Ma B. A Review of Yolo algorithm developments. Procedia Comput Sci. 2022;199:1066–73.

2. Chicco D, Jurman G. The advantages of the Matthews correlation coefficient (MCC) over F1 score and accuracy in binary classification evaluation. BMC Genomics. 2020;21(1):1–13.

3. Redmon J, Divvala S, Girshick R, Farhadi A. You only look once: Unified, real-time object detection. In: Proceedings of the IEEE conference on computer vision and pattern recognition. 2016. p. 779–88.

4. Hong S-J, Han Y, Kim S-Y, Lee A-Y, Kim G. Application of deep-learning methods to bird detection using unmanned aerial vehicle imagery. Sensors. 2019;19(7):1651.

5. Kalman RE. A new approach to linear filtering and prediction problems. 1960;

6. The MathWorks Inc. MATLAB version: 9.13.0 (R2022b) [Internet]. Natick, Massachusetts, United States: The MathWorks Inc.; 2022. Available from: https://www.mathworks.com

7. Vezza P, Libardoni F, Manes C, Tsuzaki T, Bertoldi W, Kemp PS. Rethinking swimming performance tests for bottom-dwelling fish: the case of European glass eel (Anguilla anguilla). Sci Rep. 2020;10(1):16416.
